# Immunomodulatory Potential of Kaempferol Isolated from *Peronema canescens* Jack. Leaves Through Inhibition of IL-6 Expression

**DOI:** 10.3390/ijms26073068

**Published:** 2025-03-27

**Authors:** Muhammad Ryan Radix Rahardhian, Sri Adi Sumiwi, Yasmiwar Susilawati, Muchtaridi Muchtaridi

**Affiliations:** 1 Doctoral Program in Faculty of Pharmacy, Universitas Padjadjaran, Sumedang 45363, Indonesia; muhammad20430@mail.unpad.ac.id; 2 Department of Biology Pharmacy, Sekolah Tinggi Ilmu Farmasi Yayasan Pharmasi Seamarang, Semarang 50192, Indonesia; 3 Department of Pharmacology and Clinical Pharmacy, Faculty of Pharmacy, Universitas Padjadjaran, Sumedang 45363, Indonesia; sri.adi@unpad.ac.id; 4 Department of Biology Pharmacy, Faculty of Pharmacy, Universitas Padjadjaran, Sumedang 45363, Indonesia; yasmiwar@unpad.ac.id; 5 Herbal Study Center, Faculty of Pharmacy, Universitas Padjadjaran, Sumedang 45363, Indonesia; 6 Department of Pharmaceutical Analysis and Medicinal Chemistry, Faculty of Pharmacy, Universitas Padjadjaran, Sumedang 45363, Indonesia; 7 Research Collaboration Centre for Theranostic Radio Pharmaceuticals, National Research and Innovation Agency (BRIN), Jl. Raya Bandung Sumedang KM 21, Sumedang 45363, Indonesia

**Keywords:** *Peronema canescens* Jack., kaempferol, IL-6, molecular docking, molecular dynamics, RAW 264.7

## Abstract

Sungkai leaves were selected due to their herbal medicine prevalence and documented biological activities. This study explores the immunomodulatory potential of kaempferol isolated from Sungkai (*Peronema canescens* Jack.) through a combination of in silico and in vitro methods. *P. canescens* leaves were extracted with ethanol using maceration, followed by fractionation with n-hexane, ethyl acetate, and water using a separatory funnel. Among all the fractions, the ethyl acetate fraction demonstrated the strongest inhibitory effect on IL-6 (Interleukin 6) expression, leading to further separation for the enhanced analysis of its activity. The resulting sub-fractions were purified by vacuum liquid chromatography with n-hexane and ethyl acetate gradient. Sub-fraction E was isolated through preparative thin-layer chromatography to obtain a pure compound identified as kaempferol using UV, FTIR, MS, and NMR analyses. The isolated kaempferol was then evaluated by molecular docking and molecular dynamics simulations, employing MM-PBSA (Molecular Mechanics Poisson–Boltzmann Surface Area) for binding affinity calculations. Kaempferol showed a binding affinity (ΔG) of −5.98 kcal/mol, slightly stronger than TLA (tartaric acid) (−5.90 kcal/mol). Key interactions with amino acid residues, such as Gln175, Arg182, and Arg179, were observed. Additionally, molecular dynamics simulation demonstrated that kaempferol exhibited better stability than TLA between 15 ns and 100 ns. The MM-PBSA analysis showed that kaempferol has strong van der Waals (−17.02 kcal/mol) and electrostatic interactions (−293.16 kcal/mol), with binding free energy (−17.85 kcal/mol) significantly stronger than TLA (−1.00 kcal/mol). This stability, combined with its ability to reduce IL-6 expression in vitro, highlights kaempferol’s immunomodulatory potential.

## 1. Introduction

The immune response plays a critical role in maintaining the body’s defense system against pathogens; thus, the dysregulation of this response can lead to chronic inflammatory diseases [1]. An immune response is controlled both by the direct interaction of different types of cells (lymphoid cells: B and T lymphocytes, T helper (Th) cells, and natural killer (NK) cells; myeloid cells: neutrophils, basophils, monocytes, and macrophages) and by-products of synthesis they secrete (immunoglobulins, cytokines: interleukins, colony-stimulating factors, growth factors, and interferons) [2]. The inflammatory process is a crucial component of the immune response. It is triggered by negative stimuli such as stress, tissue damage, and infections, leading histiocytes and immune cells to produce various cytokines associated with inflammation as a protective mechanism [3].

Overproduction of cytokines can provoke a dysregulated immune response and damage lung and kidney tissue, eventually progressing to multiorgan failure. Therefore, identifying safe and effective immunomodulatory strategies to manage cytokine deregulation and subsequent inflammatory responses is paramount [4]. Interleukin 6 (IL-6) is a prototypical cytokine featuring pleiotropic [5] and redundant functional activity [6]. Interleukin 6, which is promptly and transiently produced in response to infections and tissue injuries, contributes to host defense by stimulating acute phase responses, hematopoiesis, and immune reactions [7]. Under the regulation of chemokines, IL-6 induces increased trafficking of leukocytes, leading to T and B cell proliferation and differentiation [8]. IL-6 overexpression is linked to several immune-mediated diseases, such as rheumatoid arthritis, multiple sclerosis, and cancer [9].

The current treatments primarily involve synthetic IL-6 inhibitors, such as tocilizumab and sarilumab, which are monoclonal antibodies designed to block IL-6 signaling pathways. While these treatments have shown efficacy, they often have limitations, such as high treatment costs, uncomplicated infection, neutropenia, thrombocytopenia, polycythemia vera, and elevated liver enzymes [8]. An alternative treatment, such as small molecules, would offer many benefits compared to monoclonal antibodies (mAbs) therapeutic agents, such as simpler administration, and lower production costs [10]. Herbal medicines often have fewer side effects compared to synthetic drugs due to their natural composition, milder pharmacological profiles, and the use of lower doses in many cases [11]. A potential source of small molecules, herbal medicine has also gained attention as a promising alternative to synthetic drugs, offering potential solutions to overcome the limitations of current treatments [12]. Unlike synthetic drugs, herbal medicines are often associated with fewer side effects and lower costs [11]. Herbal medicines often have fewer side effects compared to synthetic drugs due to their natural composition, milder pharmacological profiles, and the use of lower doses in many cases [13]. Additionally, herbal medicines typically have lower production costs because they are often less complex to manufacture compared to other synthetic therapies [14].

*Peronema canescens* Jack. (*P. canescens*), commonly known as Sungkai, is an indigenous plant used in traditional Indonesian medicine, where it has been employed for its immunomodulatory, anti-inflammatory, and analgesic properties [15]. Several in vitro and in silico studies reported *P. canescens* leaves as an immunomodulatory [16] and anti-inflammatory [17,18]. Clinical studies have also demonstrated that *P. canescens* exhibits significant pharmacological activity, particularly as an anti-inflammatory [19]. Additionally, *P. canescens* contains a variety of bioactive compounds, including polyphenols and flavonoids, which have been shown to modulate immune responses and inhibit pro-inflammatory cytokine, such as IL-6, production [20]. The diterpenoid clerodanes present, including Peronemins B1, B2, A2, A3, B3, C1, and D1, have been identified for their potential pharmacological properties [21]. These compounds could offer a natural means to regulate the immune response and potentially inhibit IL-6 expression, making *P. canescens* a candidate for further investigation.

Recent advances in drug discovery techniques, such as isolating bioactive compounds from medicinal plants, structure elucidation, and computer-aided drug design, have paved the way for developing more targeted treatments [22]. Flavonoids like kaempferol can be isolated and evaluated for their immunomodulatory potential [23]. Molecular docking simulations allow researchers to predict the interaction between these compounds and IL-6, while molecular dynamics simulations provide insights into the stability of these interactions [24]. ADMET (absorption, distribution, metabolism, excretion, and toxicity) are fundamental pharmacokinetic properties used to evaluate the drug-likeness of a compound [25]. Furthermore, in vitro studies focused on IL-6 gene expression inhibition can validate these computational findings, aiding in the identification of potential immune response regulators.

This study focuses on *P. canescens* as a source of IL-6 inhibitors, isolating and characterizing the compounds using UV spectroscopy, mass spectrometry (MS), Fourier-transform infrared spectroscopy (FTIR), and nuclear magnetic resonance (NMR). In silico studies, including molecular docking and molecular dynamics simulations, were employed to examine the interactions of this compound with IL-6 at the molecular level, with a particular focus on hydrogen bonding and binding free energy calculations using MM-PBSA. Additionally, in vitro experiments were conducted to assess the inhibition of IL-6 gene expression in RAW 264.7 cells induced by lipopolysaccharide (LPS).

This study represents a novel approach to exploring *P. canescens* as a source of immunomodulatory agents, specifically through the isolation of kaempferol and its evaluation using in silico and in vitro methods. To our knowledge, this is one of the first studies to investigate the potential of kaempferol from *P. canescens* as an IL-6 inhibitor, which may offer a new perspective in treating immune response diseases. We believe that the findings from this study could serve as a valuable foundation for future research in this area and contribute to the development of alternative treatments for diseases associated with IL-6 overexpression. The study aims to isolate and evaluate the biological activity of kaempferol from *P. canescens* leaves as an immunomodulatory agent by using molecular docking, molecular dynamics simulation, and in vitro studies to assess its potential as an IL-6 inhibitor.

## 2. Results

### 2.1. Extract Purification and Isolation

**Figure 1:**
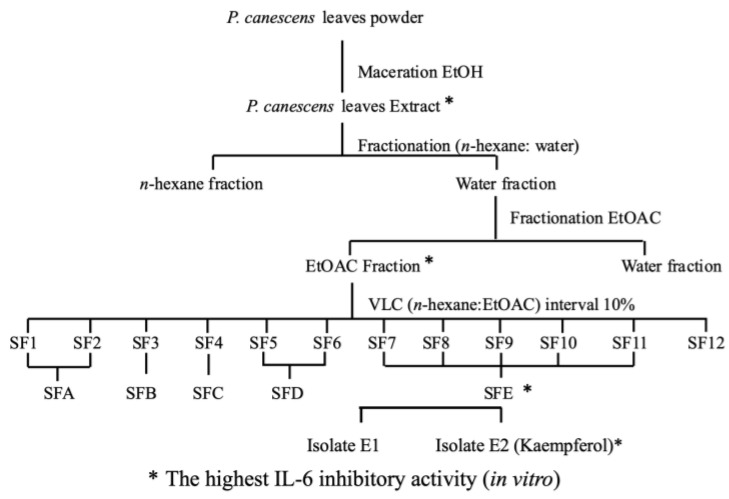
Isolation of kaempferol from *P. canescens* leaves guided by IL-6 inhibitory activity (in vitro).

Initial extraction is achieved by separating the active compounds from the leaf matrix of *P. canescens*. The method used to extract active compounds is maceration with ethanol as the solvent. As the target compounds may be non-polar to polar, the suitability of the extraction methods must be considered [26]. The fractionation of the ethanol extract was carried out using a solvent gradient system, based on polarity. This method was chosen to progressively separate fractions with varying polarity, allowing us to isolate diverse bioactive compounds including kaempferol. The fractionation process was guided by activity testing based on IL-6 inhibition at each separation step Figure 1. The first step involved the use of hot water, followed by non-polar solvents (n-hexane) and subsequently more polar solvents (ethyl acetate), enabling the separation of different classes of compounds. The application of hot water in the fractionation process aims to remove chlorophyll, which could interfere with the isolation and purification of active compounds. However, this treatment may also have facilitated the hydrolysis of glycosides. Future studies should focus on investigating the degradation pathway of kaempferol from its glycosides, particularly under different fractionation and extraction conditions. This approach is commonly employed to enrich specific bioactive molecules, such as flavonoids like kaempferol, which are typically found in more polar fractions. The yields at each stage of the fractionation process are ethanol extract 28.63%, *n*-hexane fraction 21.11%, ethyl acetate fraction 10.33%, and aqueous fraction 48.64%. Purification by fractionation process using vacuum liquid chromatography (VLC) aims to separate active compounds based on polarity level, facilitating their isolation by the preparative method of thin layer chromatography (TLC). LC-MS/MS analysis and TLC profiling confirmed the presence of kaempferol as the primary active compound in the SFE fraction derived from the ethyl acetate extract, along with other minor components. Two compounds were successfully isolated from this fraction, but only kaempferol exhibited significant bioactivity by effectively inhibiting IL-6 expression. This result highlights the success of the bioactivity-guided isolation approach, which prioritizes the identification and characterization of compounds with the strongest biological activity. The process of isolating compounds from *P. canescens* leaves is shown in Figure 1.

### 2.2. Structure Elucidation

Structural elucidation aims to determine the molecular structure of a compound to understand its biological activity and potential pharmacological applications [26,27]. This process is crucial for understanding the compound’s biological activities, pharmacological properties, and potential applications in various fields such as drug development.

The isolated compound was observed as a yellow powder. Its molecular formula was established as C_15_H_10_O_6_ according to mass spectrometry with a direct infuse (–) mode injection system, *m*/*z* 285.02 [M − H]^−^, indicating eleven degrees of unsaturation. The UV spectrum showed three absorption maximums (λmax) at 211, 268, and 337 nm, suggesting the presence of phenol as a conjugated benzene derivative chromophore and carbonyl groups. The IR spectrum showed absorption for hydroxy (3287 cm^−1^), benzene ring (1501, 1446 cm^−1^), carbonyl (1654 cm^−1^), and ether (1244 cm^−1^). Based on the two spectrums above, it can be assumed that the isolate is an aromatic group with an OH group, commonly called a phenolic compound.

**Figure 2:**
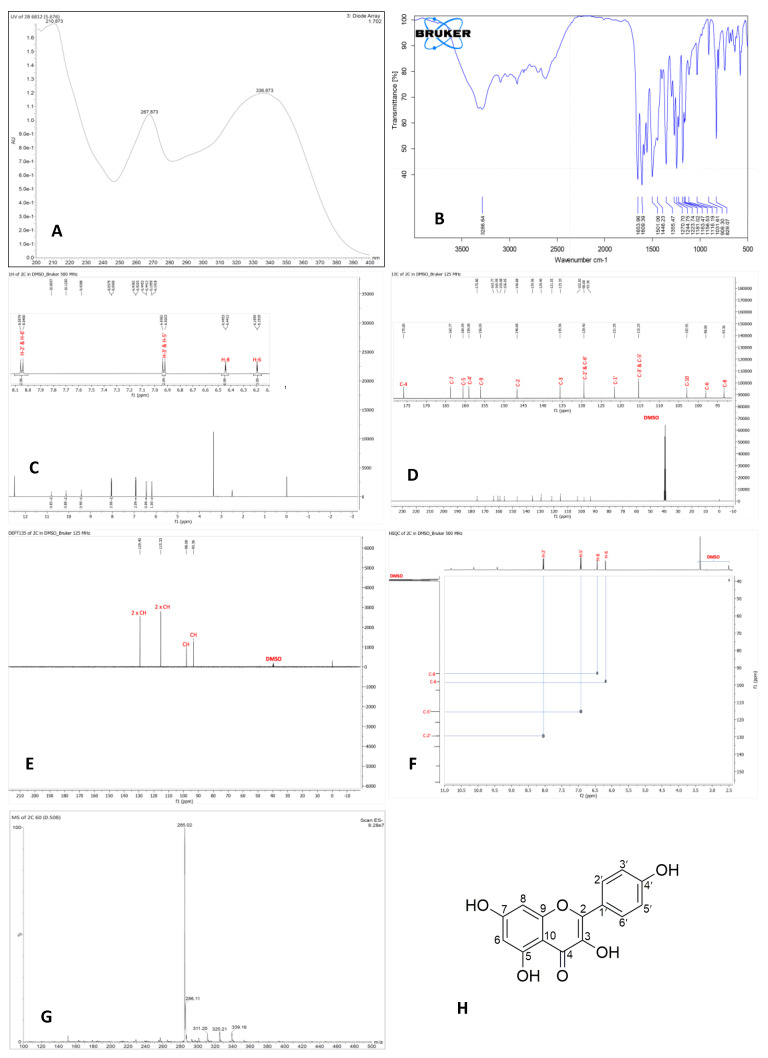
Structure elucidation of kaempferol from *P. canescens* leaves: (**A**) UV spectrum, (**B**) FTIR spectrum, (**C**) ^1^H-NMR spectrum, (**D**) ^13^C-NMR spectrum, (**E**) DEPT135, (**F**) HSQC, (**G**) MS spectrum, and (**H**) chemical structure of kaempferol.

The proton nuclear magnetic resonance (^1^H NMR) data of the isolated compound (Table 1) showed the presence of four phenolic hydroxyl singlets at δ_H_ 12.50 (5-OH), 10.80 (7-OH), 10.13 (4′-OH), and 9.43 (3-OH); a pair of overlapped ortho-coupled aromatic doublets at δ_H_ 8.05 (H-2′/H-6′) and 6.93 (H-3′/H-5′) with coupling constants of 9.0 Hz, which corresponds to four aromatic protons in ring B as a characteristic of the 1′,4′-disubtituted flavone [28]; and a pair of meta-coupled aromatic doublets at δ_H_ 6.44 (H-8) and 6.19 (H-6) with coupling constants of 2.0 Hz. From the ^1^H-NMR data, it can be assumed that the isolated compound has two phenolic structures. Moreover, the deshielded hydroxyl proton showed the chelatogenic group in its structure as in flavonoids [29]. One-dimensional and two-dimensional NMR analysis must be carried out to ascertain the structure’s shape Table 1 and Figure 2. In addition, the ^13^C and DEPT NMR data of the isolated compound (Table 1, Figure 2) showed the presence of one chelating carbonyl carbon at δ_C_ 175.8 (C-4); six oxygenated carbon sp2 at δ_C_ 163.8 (C-7), 160.6 (C-5), 159.1 (C-4′), 156.0 (C-9), 146.7 (C-2), and 135.6 (C-3); six protonated carbon sp2 at δ_C_ 129.4 (C-2′/C-6′), 115.3 (C-3′/C-5′), 98.1 (C-6), and 93.4 (C-8); and two non-protonated carbon sp2 at δ_C_ 121.6 (C-1′) and 102.9 (C-10). Thus, a total of fifteen carbon signals were observed in ^13^C and DEPT NMR spectral data. The eight degrees of unsaturation were accounted for from the NMR data, and the remaining three degrees of unsaturation consisted of flavonol structure. Furthermore, comparing the ^1^H and ^13^C NMR data of isolated compounds with those of kaempferol [28] revealed that the structures of those two compounds are similar. Therefore, it can be concluded that the isolated compound was identified as kaempferol or namely 3,5,7-trihydroxy-2-(4′-hydroxyphenyl) chromene-4-one.

### 2.3. Molecular Docking Simulation

**Figure 3:**
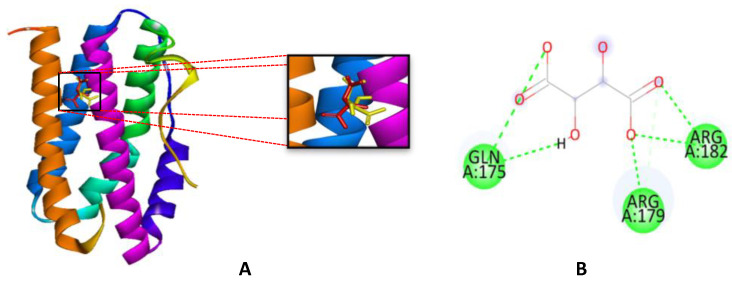
Overlay of docking and crystallographic (red) before docking (yellow) after docking (**A**), Interaction of TLA with amino acid residues from IL-6 (**B**).

Docking is the molecular modeling technique that helps predict protein interaction with ligands [30]. The Autodock 4.2 software was applied for molecular docking studies using Human IL-6 (PDB ID: 1ALU) [31]. Although the PDB ID 1ALU is associated with the IL-6 cytokine, it is used as a model for the interaction with the receptor [18]. To validate the docking procedure, the ligand TLA (Tartaric Acid) [31], complexed and crystallized at 1ALU, was subjected to redocking. The outcome of the redocking process is visually presented in Figure 3, depicted in red, while the position derived from the crystallographic result is represented in green. The legitimacy of the docking procedure on the redocked ligand’s location and bond alignment with those observed during the crystallization process is supported by an RMSD value of two [32].

To validate the AutoDock4 application, the results of redocking TLA revealed hydrogen bond interactions with the residues Arg 179, Gln 175, and Arg 182 [18], Arg 182, Arg179, and Gln 175 [31]. This interaction profile further affirms the accuracy and reliability of the AutoDock4 application in reproducing the binding configuration of TLA with the IL-6 binding site. A molecular docking simulation was carried out with kaempferol and TLA as ligand models. The docking procedure was aided by the use of the previously specified parameters. It was 2,500,000 iterations using 100 conformations with genetics algorithm runs. This strategy leverages the established effectiveness of the genetic algorithm method to provide a robust generation of different samples for the investigation of ligand conformation [18].

Molecular docking simulation allows the assessment of ligand–receptor interaction affinity by determining the binding energy [33]. The binding affinity (∆G) of TLA is −5.90 kcal/mol (cluster 93 out of 100), which is greater than the ∆G of kaempferol −5.98 kcal/mol (cluster 91 out of 100), indicating that the kaempferol has a stronger binding than TLA. According to [34], the binding affinity of the IL-6–kaempferol complex is reported as −5.6 kcal/mol. This study demonstrates a stronger interaction between kaempferol and its target compared to previous research [34]. Although the difference in binding affinity is small, it is still significant, as even slight variations can impact the ligand’s ability to stabilize its bond with the target protein. It is recommended that future studies include multiple replicates of docking simulations to enable statistical analysis of binding energy values.

The inhibition constant (Ki) of TLA is 47.35 μM and kaempferol is 41.28 μM. Ki is an essential parameter in molecular docking that indicates how strongly the ligand binds to and inhibits the target protein. Lower Ki indicates higher affinity and greater potency as an inhibitor [35]. The lower Ki value proves that kaempferol has a higher affinity than TLA, emphasizing its role in identifying binding pockets.

**Figure 4:**
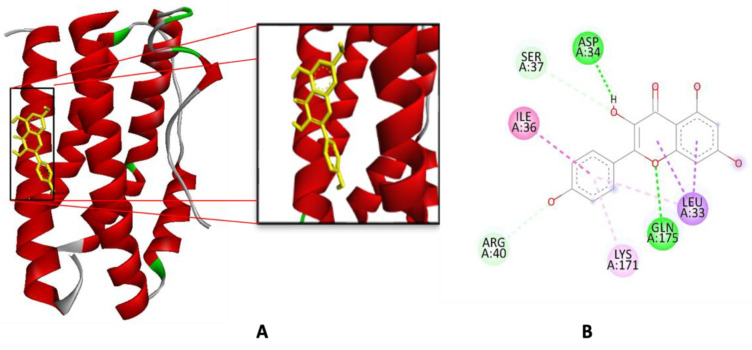
Interaction of kaempferol with amino acid residues of IL-6: (**A**) 3D representation and (**B**) 2D representation.

The interaction of the TLA complex with IL-6 (PDB ID: 1ALU) involves several hydrogen bond interactions at key amino acid residues, including Gln175, Arg182, and Arg179 as seen in Table 2 and Figure 4 Similarly, when interacting with kaempferol, the complex forms hydrogen bonds at Gln175 and Asp34. Additionally, there are other interactions, such as Pi-Sigma bonding with Leu33, Pi-alkyl bonding with Lys171, and Pi-amide bonding with Ile36. These bonds play a critical role in stabilizing the kaempferol-IL-6 complex.

### 2.4. Pharmacokinetic and Toxicity Prediction

Pharmacokinetic and toxicity predictions use Deep PK, accessed through https://biosig.lab.uq.edu.au/deeppk/ (accessed on 22 January 2024). To obtain pharmacokinetic and toxicity data from the in vivo or preclinical stage is time-consuming and expensive. Deep-PK will predict the properties of ADMET through computational approaches, a deep learning-based platform in pharmacokinetics and toxicity prediction as well as implementing Graph Neural Networks [36]. The ADMET of kaempferol (Table 3) from absorption with human colorectal adenocarcinoma cells (Caco-2), distribution using the blood–brain barrier (BBB), metabolism in CYP2D6, excretion with total clearance, and toxicity with Ames prediction [37].

### 2.5. Molecular Dynamics Simulation

Molecular dynamics simulation was employed to observe the characteristics and interaction of the kaempferol complex with IL6. This involved observing the behavior of the ligand–receptor complex over a 100 ns period, adhering to specific temperature and pressure conditions [32]. The molecular dynamics simulation of kaempferol was carried out for 100 ns using AMBER22, and the results were analyzed using RMSD and RSMF charts [38].

**Figure 5:**
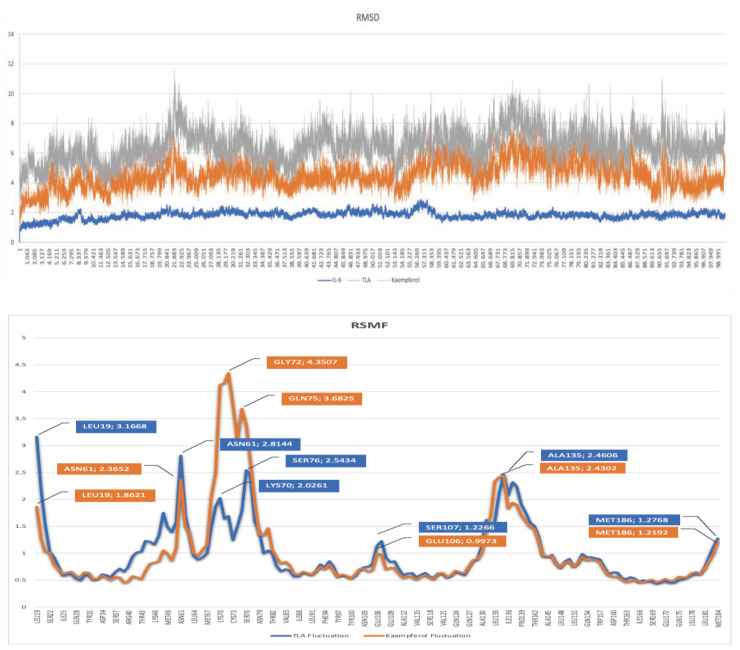
RMSD value of kaempferol and TLA as ligands, and IL-6 as a receptor in the complex from molecular dynamics (**A**). RMSF kaempferol and TLA from molecular dynamics (**B**).

Root mean square deviation (RMSD) is a commonly used metric in molecular dynamics simulations to assess the structural stability of a system over time. It measures the average distance between atoms in a structure compared to a reference structure, usually the starting or minimized conformation. RMSD can help determine if a system reaches equilibrium or undergoes significant conformational changes during a simulation [39]. The ligand–IL-6 interaction was parameterized using the ff14SB, GAFF, and AM1-BCC partial charge calculation [32]. The average distance that atoms traveled away from the reference structure is shown by the RMSD values of kaempferol, TLA, and IL-6 during the molecular dynamics simulations in Figure 5. The plot represents the stability and conformational changes in the ligand (kaempferol and TLA) and the receptor (IL-6) over a simulation period of 100 ns. The RMSD values for the ligands are calculated relative to their initial positions, while the RMSD values for the protein reflect the overall structural stability. The RMSD values presented in Figure 5 represent the stability of the protein–ligand complexes (kaempferol-IL-6 and TLA-IL-6) during the molecular dynamics simulations.

Interestingly, although kaempferol forms stronger hydrogen bonds with IL-6, as indicated by its hydrogen bond acceptors and donors Table 4, the RMSD analysis reveals higher fluctuations in the kaempferol-IL-6 complex and TLA-IL-6 (Figure 5). This phenomenon suggests that the stronger binding of kaempferol may induce greater flexibility in the receptor’s binding pocket, a dynamic behavior commonly observed in protein–ligand interactions involving highly flexible ligands or strong binding energies [40]. The RMSF analysis reveals similar patterns of residual fluctuations between kaempferol and TLA, with both compounds showing notable fluctuations at several key residues. The residues exhibiting significant RMSF for TLA include Leu19, Asn61, Lys70, Ser76, Ser107, Ala135, and Met186 Figure 5. In contrast, kaempferol shows significant fluctuations at Leu19, Asn61, Gly72, Gln75, Glu106, Ala135, and Met186. Additionally, the hydrogen bond analysis indicates that kaempferol predominantly forms hydrogen bonds with the residue Phe98, demonstrating a fraction of 0.20, while TLA has significant interactions with Arg155, with a fraction of 0.09. These findings highlight a distinct difference in the hydrogen bonding profiles between kaempferol and TLA, particularly concerning the Phe98 residue in kaempferol, suggesting that despite some similarities in fluctuation patterns, the two compounds interact differently at the molecular level. This information is crucial for understanding these compounds’ binding dynamics and potential efficacy in therapeutic applications.

MM-PBSA (Molecular Mechanics Poisson–Boltzmann Surface Area) is a widely used method in molecular dynamics simulations to estimate the binding free energy of biomolecules, such as proteins and ligands [39]. The binding energy was then computed using the MM-PBSA method to predict the binding affinity of kaempferol-IL6 complex Table 4. The MM-PBSA approach is regarded as more advanced for calculating binding free energy than MM-GBSA [41]. The results for TLA show a modest total binding free energy (∆G_TOTAL_) of −1.00 kcal/mol, indicating a weak interaction Table 5. The negative values of ∆G_EL_ and ∆G_VDW_ suggest that attractive electrostatic and Van der Waals interactions are present.

### 2.6. Immunomodulatory Activity by Inhibition of IL-6 Gene Expression

**Figure 6:**
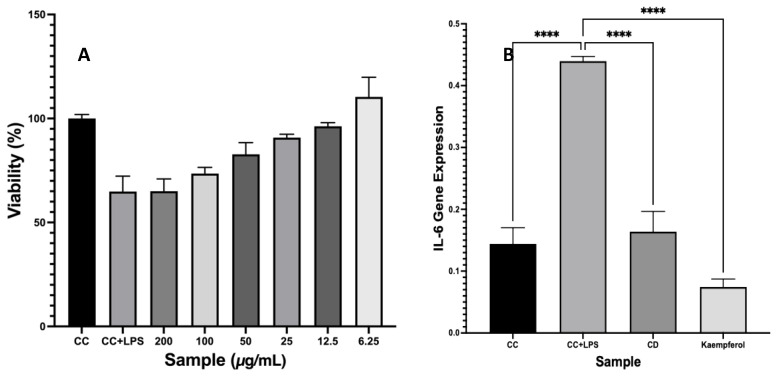
Immunomodulatory activity from kaempferol to inhibit IL-6 gene expression induced LPS. Viability of kaempferol (**A**), IL-6 gene expression of kaempferol (**B**). Values are mean SD of three independent experiments. **** *p* < 0.0001.

Kaempferol concentrations of 25 μg/mL and 12.5 μg/mL maintain a cell viability greater than 80%, suggesting that these concentrations are non-toxic and suitable for further experimentation. Given that a viability threshold above 80% is generally accepted as an indicator of safety in cytotoxicity assays, kaempferol derived from *P. canescens* leaves can be deemed safe for subsequent tests (Figure 6). A concentration of 12.5 μg/mL was selected, which yielded a remarkable viability value of 98.17 ± 2.94. This high viability indicates that the cells remained largely unaffected by this concentration of kaempferol, thus affirming its potential as a safe therapeutic agent. It is essential to determine the appropriate concentration of kaempferol, especially when testing its effects on immune responses, such as inhibiting IL-6 gene expression in RAW 264.7 cells.

The IL-6 inhibitory activity of each fraction and isolate was evaluated and expressed as a percentage inhibition in IL-6 expression at a concentration of 12.5 µg/mL. The ethyl acetate fraction (FEA) showed the highest inhibitory activity with a 45% reduction, followed by the n-hexane fraction (FNH) with 5% inhibition. In contrast, the aqueous fraction (WF) exhibited a −37.5% change, indicating no inhibitory effect. Sub-fraction E (SFE) achieved 50.02% inhibition, while kaempferol, isolated from the SFE fraction, demonstrated a significantly higher inhibition of 84.09% under the same conditions.

## 3. Discussion

### 3.1. Extract Purification and Isolation

Extraction is a critical step in isolating bioactive compounds from the leaf matrix of *P. canescens*. In this research, the maceration method using 96% ethanol as a solvent was employed to extract active compounds. Kaempferol, a flavonoid known for its potent antioxidant [42] and immunomodulatory properties, was successfully isolated from *P. canescens* in this study. This compound has been the subject of significant research due to its wide range of pharmacological activities [23]. Studies have demonstrated that kaempferol exerts its biological effects by modulating various signaling pathways, making it a promising candidate for drug development [43].

### 3.2. Structure Elucidation

The physical and chemical properties of the isolated pure compound fully correspond with the literature values for kaempferol, including color, UV [28,42], FTIR analysis [28,44,45], ¹H- and ¹³C-NMR spectra [28,46], and molecular weight [23,44,47,48].

### 3.3. Molecular Docking Simulation

Molecular docking is a computational technique used to predict the interaction between a small molecule (ligand) and a target protein, providing insights into the binding affinity and orientation of the ligand within the protein’s binding site [49]. The primary goal of molecular docking is to identify ligands that can effectively bind to a specific protein target, leading to potential therapeutic applications by inhibiting or modifying the protein’s function. In this study, molecular docking was employed to evaluate the binding affinity of kaempferol, a flavonoid compound isolated from *P. canescens*, and compare its interaction with the IL-6 protein (PDB ID: 1ALU) against TLA. The results of the molecular docking simulation revealed that the binding affinity (ΔG) of the TLA was −5.90 kcal/mol (cluster 93 out of 100). In contrast, the binding affinity of kaempferol was slightly lower at −5.98 kcal/mol (cluster 91 out of 100). A lower ΔG value indicates stronger binding affinity between the ligand and the target protein, suggesting that kaempferol forms a more stable complex with IL-6 than TLA. Binding affinity is a crucial parameter in molecular docking studies, as it represents the free energy change (ΔG) associated with the ligand binding to the protein [50]. A more negative binding affinity (lower ΔG value) corresponds to a more stable interaction between the ligand and the protein, indicating that the ligand binds more tightly to the protein [51]. In contrast, a less negative (higher) ΔG value reflects a weaker interaction, meaning the ligand binds less effectively to the protein. In this study, the binding affinity of kaempferol (−5.98 kcal/mol) being lower than that of the TLA (−5.90 kcal/mol) suggests that kaempferol has a stronger and more favorable interaction with IL-6.

The stronger binding affinity of kaempferol may also be explained by the molecular structure of kaempferol, which allows it to form additional non-covalent interactions, thereby enhancing its overall binding stability. Kaempferol’s flavonoid structure includes multiple hydroxyl groups capable of forming hydrogen bonds, and its planar aromatic rings facilitate hydrophobic and pi-stacking interactions [23]. These structural features make kaempferol a more effective ligand for stabilizing the IL-6 complex. Comparing these results with similar studies, previous research has shown that flavonoids, including kaempferol, exhibit strong binding affinities with various protein targets due to their ability to form multiple non-covalent interactions. For instance, a study by [52] reported a binding affinity of −5 kcal/mol for kaempferol with IL-6 while −6.4 kcal/mol for kaempferol with IL-6 was observed by Hossain et al. [53] both slightly higher than the value found in this study. This difference may be attributed to variations in docking protocols or the specific conformations of the protein and ligand used in the simulation. However, the overall trend remains consistent: kaempferol exhibits a robust binding affinity, confirming its potential as an inhibitor of IL-6.

The inhibition constant (Ki) is a crucial parameter in molecular docking that reflects the binding strength between a ligand and its target protein. A lower Ki value indicates a stronger interaction and a higher binding affinity of the ligand for the target, implying greater inhibitory potency. Conversely, a higher Ki value suggests weaker binding and lower inhibitory effectiveness. In this study, the molecular docking results for the IL-6 protein (PDB ID: 1ALU) revealed that the TLA has an inhibition constant of 47.35 μM, while kaempferol showed a lower Ki of 41.28 μM. This smaller Ki value for kaempferol indicates that it binds more tightly to IL-6 than TLA, suggesting that kaempferol is a more potent inhibitor of this protein. The differences in Ki values can be attributed to several factors, including the molecular structure of the ligands, the type and strength of interactions with the target protein, and the conformational flexibility of the binding site [54].

The interaction analysis further supports kaempferol’s superior binding. Both kaempferol and TLA form hydrogen bonds with the key residue Gln175, a critical point of interaction that stabilizes the ligand–protein complex. However, kaempferol forms additional interactions, including a hydrogen bond with Asp34 and non-covalent interactions, such as pi-sigma bonding with Leu33, pi-alkyl bonding with Lys171, and pi-amide bonding with Ile36. These additional interactions contribute to the enhanced stability of the kaempferol-IL-6 complex. In contrast, TLA only forms hydrogen bonds with Gln175, Arg182, and Arg179, lacking the diverse range of interactions seen with kaempferol. According to [55], the IL-6 (1ALU) complex with kaempferol forms hydrogen bonds with residues Gln99 and Val160 and hydrophobic interactions with His116, Phe162, and Val193. Notably, the complex also features a π-π interaction involving Phe162, further enhancing the stability and specificity of the binding. According to [34], additional residues involved in the IL-6 (1ALU) and kaempferol interaction include Glu95, Gln116, Lys120, Pro139, and Pro141. These interactions contribute to the complex’s overall structural and functional integrity, highlighting the importance of specific residue interactions in modulating the activity of IL-6 when bound to kaempferol. This detailed understanding of the molecular interactions provides valuable insight into how kaempferol could potentially inhibit IL-6 activity, offering a framework for developing therapeutic strategies targeting IL-6-related pathways.

### 3.4. Pharmacokinetic and Toxicity Prediction

Kaempferol absorption was evaluated using human colorectal adenocarcinoma (Caco-2) cells, with a log Papp value of −5.26. Caco-2 cells are commonly used as an in vitro model to predict human oral drug absorption [56]. A compound is considered to have high Caco-2 permeability if its Papp exceeds 8 × 10^−6^ cm/s. Since kaempferol falls below this threshold, its absorption through the intestinal barrier is likely low. Similar findings indicate that kaempferol undergoes rapid metabolism and efflux by intestinal transporters, such as P-glycoprotein, further limiting its systemic absorption [57]. Various approaches have been explored to overcome these limitations, including nanoformulations [58]. The blood–brain barrier (BBB) permeability of kaempferol, with a logBB value of −2.75, suggests poor penetration into the brain. A logBB > 0.3 is typically associated with good BBB permeability, while a value below −1 indicates poor distribution to the brain. Therefore, kaempferol is unlikely to cross the BBB significantly, limiting its potential for central nervous system (CNS) activity, thus reducing the risk of CNS-related side effects. Kaempferol is not metabolized by the cytochrome P450 enzyme CYP2D6, which plays a crucial role in drug metabolism. The lack of CYP2D6 metabolism reduces the likelihood of drug–drug interactions and suggests that kaempferol may have a relatively stable metabolic profile. However, further studies would be needed to assess interactions with other cytochrome P450 enzymes. The total clearance of kaempferol is predicted to be 5.81 log mL/min/kg. Clearance is a key pharmacokinetic parameter determining how long a drug remains in the bloodstream. Kaempferol’s clearance suggests it is eliminated relatively quickly from the body. Kaempferol was predicted to be non-toxic based on the Ames test, which assesses mutagenic potential using bacterial strains [25]. The absence of genotoxicity indicates that kaempferol is unlikely to pose a significant mutagenic risk, supporting its potential as a safe therapeutic compound. The ADMET analysis of kaempferol reveals low absorption and poor BBB permeability but suggests a favorable safety profile with no significant toxicity concerns. These findings were in line with similar studies on kaempferol ADMET [53].

### 3.5. Molecular Dynamics Simulation

Molecular dynamics (MD) simulations are a computational technique used to study the physical movements of atoms and molecules over time. By simulating these dynamics, researchers can investigate the stability, conformational changes, and interactions between ligands and their target proteins under physiological conditions. MD provides valuable insights into the time-dependent behavior of protein–ligand complexes, such as binding stability, fluctuations in atomic positions, and the strength of molecular interactions. In this study, MD simulations were employed to evaluate the stability of the kaempferol-IL-6 (PDB ID: 1ALU) complex for 100 ns. The results of the MD simulation revealed that kaempferol exhibits superior stability within the IL-6 binding site, beginning at 15 ns (15,000 ps) and continuing through the remainder of the 100 ns simulation. Comparing these findings with similar studies on kaempferol, the results are consistent with previous studies, reinforcing its potential as a stable inhibitor in the IL-6 pathway [59].

This is indicated by the lower root mean square deviation (RMSD) values for kaempferol, which ranged from 0.5 to 1 Å. RMSD measures the average distance between atoms of the protein–ligand complex over time and indicates structural stability [24]. Lower RMSD values imply greater stability, as smaller deviations suggest that the ligand remains more tightly bound to the protein and undergoes fewer conformational changes during the simulation [60]. The enhanced stability of kaempferol can be attributed to several factors. First, the molecular structure of kaempferol enables it to form more favorable and consistent interactions with key residues in the IL-6 binding pocket [61]. As previously discussed, kaempferol forms hydrogen bonds with Gln175 and Asp34 and pi-alkyl and pi-sigma interactions with Lys171 and Leu33, respectively. These interactions are essential for maintaining the structural integrity of the protein–ligand complex, reducing fluctuations, and contributing to kaempferol’s overall stability [62]. The RMSD values for kaempferol and TLA are similar, measuring around 0–2 Å. The IL-6 flexibility is stable when interacting with kaempferol and TLA, as the protein remains folded and does not alter significantly during dynamic simulations [63].

Trajectory analysis of the simulation results was also carried out on the Root Mean Square Fluctuation (RMSF) value. The RMSF value is a condition of fluctuations in the amino acid residues that comprise the receptor during the simulation process. Fluctuations represent the flexibility of the amino acid residues. RMSF is used to measure the flexibility of specific atoms, typically the backbone atoms of residues, in a molecular system of a simulation [39]. The RMSF analysis establishes the stability pattern to study protein and amino acid composition changes that occur during the interaction of the kaempferol and TLA complexes. RMSF focuses on the protein’s complex region and its fluctuating amino acid residues. During the MD simulations, High RMSF values indicate regions of the molecule that are highly flexible (e.g., loops or termini). Low RMSF values indicate more stable regions (e.g., alpha-helices or beta-sheets) [32]. However, the positive value of ∆E_GB_ (36.68 kcal/mol) indicates significant solvation penalties, which diminish the overall binding affinity. The negligible surface energy contribution (∆E_SURF_ = −0.19 kcal/mol) further reflects the minimal impact of surface area on solvation effects for TLA.

In contrast, kaempferol exhibits a significantly more favorable binding free energy (∆G_TOTAL_) of −17.85 kcal/mol. The significant negative values for ∆G_VDW_ (−17.02 kcal/mol) and ∆G_EL_ (−293.16 kcal/mol) indicate strong, attractive interactions, suggesting that kaempferol forms robust binding interactions with the target. However, the positive ∆E_GB_ (295.06 kcal/mol) indicates considerable solvation penalties, similar to TLA. The relatively larger contribution from the solvation-free energy (∆G_SOLV_ = 292.33 kcal/mol) suggests that while kaempferol interacts favorably, the solvation effects are also substantial. The MM-PBSA analysis indicates that kaempferol demonstrates a more favorable binding affinity than TLA, primarily due to its stronger Van der Waals and electrostatic interactions. However, both compounds experience substantial solvation penalties that affect their overall binding energies. These insights can guide further investigations into the molecular mechanisms underlying their interactions and potential therapeutic applications.

### 3.6. Immunomodulatory Activity by Inhibition of IL-6 Gene Expression

The necessity of conducting viability testing in RAW 264.7 cells with LPS induction is highlighted as a critical step in defining concentrations for future testing. Lipopolysaccharides (LPSs) are a standard method to simulate an inflammatory response, allowing researchers to evaluate the anti-inflammatory effects of kaempferol. Subsequently, a 25 μg/mL concentration was employed to investigate the inhibition of IL-6 gene expression. The choice of this concentration is based on its likely balance of efficacy and safety, as established in the initial viability tests. The findings from [16] support this approach. Their study employed an MTT assay to assess the viability of RAW 264.7 cells treated with *P. canescens* ethanol extract at various concentrations (1, 10, and 100 μg/mL), reporting viability values of 128.10 ± 2.85, 128.83 ± 5.79, and 135.82 ± 0.81, respectively. The kaempferol did not adversely affect cell viability, which reinforces the idea that kaempferol is a promising candidate for further exploration in anti-inflammatory therapies.

Kaempferol has the best IL-6 gene expression inhibition activity compared to cell control and DMSO. Based on these results, kaempferol is the active compound in the search for immunomodulatory active compounds in inhibiting IL-6 gene expression from *P. canescens* leaves. The IL-6 gene expression analysis demonstrates the impact of kaempferol on inhibiting the inflammatory response in RAW 264.7 cells. IL-6 is a key pro-inflammatory cytokine, and its expression is typically upregulated in the presence of inflammatory stimuli like lipopolysaccharides (LPSs). The data shows gene expression levels under various conditions: control cells, LPS-induced cells, DMSO control, and kaempferol-treated cells. The baseline IL-6 gene expression levels in untreated control cells ranged between 0.116 and 0.168. These values represent normal IL-6 expression without any external inflammatory stimuli. Upon induction with LPS, IL-6 gene expression significantly increased, with values between 0.431 and 0.446. This notable increase highlights the inflammatory response triggered by LPS, as it mimics bacterial infection and strongly induces cytokine production, including IL-6. In the DMSO control group, the IL-6 gene expression values ranged from 0.140 to 0.201. These levels are somewhat elevated compared to the untreated control but significantly lower than in the LPS-induced group. DMSO, a common solvent used in cell-based assays, did not appear to significantly affect IL-6 expression, indicating that it is suitable for use as a control. The cells treated with kaempferol exhibited the lowest IL-6 gene expression levels, ranging from 0.061 to 0.087. This represents a substantial reduction in IL-6 expression compared to the LPS-induced group. The decrease in expression suggests that kaempferol effectively inhibits the pro-inflammatory IL-6 response. The average IL-6 expression in the kaempferol-treated group is lower than both the control and DMSO control groups, indicating a strong anti-inflammatory effect of kaempferol.

Kaempferol significantly reduces IL-6 gene expression compared to LPS-induced cells. In the LPS-induced group, IL-6 expression was increased nearly three to four times compared to the control group, reflecting a typical inflammatory response. However, in the presence of kaempferol, IL-6 levels were reduced to levels even lower than the untreated control cells. These data suggest that kaempferol possesses potent anti-inflammatory properties, as it can downregulate IL-6 expression even under strong inflammatory conditions. Its ability to inhibit IL-6 may be related to its potential as a therapeutic agent for chronic inflammation, such as autoimmune disorders or inflammatory diseases. According to [1], kaempferol demonstrates a broad neuroprotective effect by regulating multiple pro-inflammatory signaling pathways, such as NF-κB, p38MAPK, AKT, and the β-catenin cascade. Kaempferol inhibited the activation and nuclear translocation of two key transcription factors, STAT3 and NF-κB, which collectively regulate COX-2 induction in response to IL-6 [2]. Kaempferol functions as a scavenger of free radicals and superoxide radicals while also maintaining the activity of key antioxidant enzymes, including catalase, glutathione peroxidase, and glutathione-S-transferase [3].

The FEA (ethyl acetate fraction) exhibited the highest inhibitory activity on IL-6 expression, reducing IL-6 levels by 45%. This suggests that FEA contains a significant concentration of bioactive compounds, particularly those capable of modulating inflammatory pathways. The FNH (n-hexane fraction) demonstrated a much lower inhibition of 5%, likely due to the predominance of non-polar compounds with limited interaction or binding affinity for IL-6 regulatory sites. Non-polar compounds, such as fatty acids or terpenoids, are less likely to exhibit strong anti-inflammatory effects compared to flavonoids. In contrast, the WF (aqueous fraction) displayed a negative inhibition percentage (−37.5%), indicating an increase in IL-6 expression. This suggests that the WF may contain inactive or potentially pro-inflammatory compounds, such as carbohydrates or other primary metabolites, that do not contribute to IL-6 suppression. Such compounds might also interfere with the binding or activity of inhibitory molecules. Interestingly, the SFE (Sub-fraction E), derived from the ethyl acetate fraction, demonstrated a 50.02% inhibition of IL-6 expression at 12.5 µg/mL, reflecting the enrichment of bioactive compounds, particularly kaempferol, through the fractionation process. When isolated, kaempferol achieved an even higher inhibition of 84.09%.

The higher inhibitory activity of pure kaempferol suggests that the other compounds present in the SFE fraction may modulate its overall bioactivity. While kaempferol is the primary contributor to the observed activity, other compounds present in SFE may exert neutral, synergistic, or antagonistic effects. This study employed a bioactivity-guided isolation approach to identify and characterize kaempferol as the active compound responsible for IL-6 inhibition. However, the potential contribution of the other compounds in SFE warrants further investigation. Although the IL-6 inhibition activity is primarily attributed to kaempferol based on in vitro and in silico experiments, it is important to note that the other metabolites present in the active fraction may contribute to the observed biological effects. Future studies should investigate the potential synergistic or combined effects of the other compounds in *P. canescens* leaves to gain a more comprehensive understanding of its immunomodulatory mechanism. Further research is needed to validate the in vitro and in silico findings on the immunomodulatory activity of kaempferol and to explore its mechanisms at the in vivo level. Future studies should include pathway analyses, such as Western blot assays for NF-κB and MAPK signaling pathways, to elucidate its molecular targets in IL-6 regulation, as well as molecular dynamics simulation to better understand the stability and interaction of kaempferol with its molecular targets. These experiments would provide deeper insights into its immunomodulatory mechanisms and further support its potential as a therapeutic agent.

## 4. Materials and Methods

### 4.1. Sample Preparation

The *P. canescens* leaves were collected from the district Padang Pariaman in west Sumatera and verified at the Herbarium Jatinangorriensis at the Padjadjaran University in West Java, with the identification number 05/LBM/IT/II/2021.

### 4.2. Extraction, Fractionation, and Isolation

*P. canescens* leaf powder was weighed in 3600 g and extracted using 96% ethanol solvent using the re-maceration method [64]. The powder was soaked for 5 days. After 5 days, it was separated from the solvent, added to a new 96% ethanol solvent, and soaked for an additional 3 days. The liquid extract obtained was then concentrated with a vacuum rotary evaporator, followed by a water bath at a temperature of 50^0^C until a viscous extract was obtained. The yield of the extract was calculated. The ethanol extract of *P. canescens* leaves was dissolved in hot water. The water fraction was then put into a separate funnel added to the n-hexane, and then shaken. After that, it was separated between the water and n-hexane phases and repeated until the n-hexane phase was clear. The water fraction was put into a separation funnel, and ethyl acetate was added, and then shaken and separated between the water and ethyl acetate phases, repeated until the ethyl acetate phase was clear. When performing the fractionation process, each solvent was repeated to obtain a greater yield of compounds. After all fractions were obtained, the liquid fraction was concentrated with a rotary vacuum evaporator, and the fraction yield was calculated [65].

The fractionation and isolation process were then carried out by VLC (vacuum liquid chromatography) and PTLC (preparative thin-layer chromatography). VLC was carried out with silica gel G 60 and gradient elution using the mobile phase of n-hexane-ethyl-methanol with various comparisons of 100 mL each. The results from VLC obtained 12 fractions, which were assigned sub-fraction codes SF1 to SF12. The grouping of factions was carried out after being identified with the TLC for the 12 factions. The factions with similar Rf values from TLC were combined until 5 sub-fractions were obtained, and sub-fraction codes were assigned from SFA to SFE. A quantitative analysis using LC-MS/MS and qualitative analysis through TLC were conducted to characterize the active compounds in the SFE fraction. Preparative TLC guided by bioactivity was used to separate and isolate bioactive compounds.

### 4.3. Elucidation of the Compound Structure

The spectroscopic methods used were UV, IR spectroscopy, FTIR (Bruker ALPHA FTIR Spectrometer, Bruker Corporation, Billerica, MA, USA) (KBr System), 1-D and 2-D NMR Avance III Bruker 500 MHz Liquid System, Bruker Corporation, Billerica, MA, USA (^1^H and ^13^C-NMR with DEPT technique), and UPLC-MS/MS (ACQUITY UPLC H-Class System with PDA Detector and XEVO-TQD MS, Waters Corporation, Milford, MA, USA) (Direct Infuse System) [38].

### 4.4. Molecular Docking Simulation

Molecular docking simulation was performed using Autodock 4.2 [66]. The 3D structures of TLA and kaempferol were optimized using Chem3D Ultra 8.0 and the semi-empirical computational technique MM2. TLA was selected as a control ligand because it is co-crystallized with IL-6 in the PDB structure (PDB ID: 1ALU). The calculation was performed by maximizing the geometry at the 3D structure’s minimal energy. The pdbqt format was used to tether each ligand to the receptor, grid box size 60 × 60 × 60, spacing 0.375 (À), x, y, z coordinates respectively −7.677, −12.743, 0.007. The interactions with biomacromolecules were solid, and each ligand was stable. Discovery Studio Visualizer was used to view the amino acid residue interactions, including hydrogen bonds, hydrophobic interactions, and bond lengths [18]. An analysis was performed to evaluate the binding energy, interactions between the ligand and key amino acid residues, and the inhibition constant (Ki).

### 4.5. Molecular Dynamics Simulation

The molecular dynamics simulations were carried out using the AMBER 22 software. The parameterization of ligands and IL-6 was performed using ff14SB and GAFF force field. The system’s total charge was neutralized with counterions (Na^+^/Cl^−^) and solvated using a TIP3P water model with a 10 Å buffer around the protein–ligand complex. Periodic boundary conditions were applied to ensure complete solvation throughout the simulation. The charge of the ligands was balanced through restricted electrostatic potential, and topology files played a crucial role in supporting the simulation process, including heating, equilibration, and production runs. To ensure compatibility with the designated force field, a heating function was applied for both ligands and receptors before initiating the production phase of the molecular dynamics simulation. This involved a three-step heating process, increasing the system temperature from 0 to 310 K, and pressure (1 atm) at regular intervals for physiological conditions. Subsequently, system stability was achieved through equilibration, reaching a steady state before generating the molecular dynamics simulation [32]. The simulation duration was 100 ns, and the analysis tools used were root mean square deviation (RMSD), Root Mean Square Fluctuation (RMSF), MM-PBSA, and hydrogen bond. Trajectory analysis was performed using the xmgrace module in the AMBER 22 software [39].

### 4.6. Prediction of ADMET

The ADMET (absorption, distribution, metabolism, excretion, and toxicity) program can be accessed at https://biosig.lab.uq.edu.au/deeppk/ (accessed on 22 January 2024) [36]. The structure was converted to SMILE format using PubChem. The structure was downloaded using the canonical SMILE.

### 4.7. Evaluation of Cell Viability in RAW 264.7 Macrophage Cells

The measurement was conducted using an enzyme-linked immunosorbent assay (ELISA). Each sample was weighed and dissolved incrementally in DMSO with vortex mixing until fully dissolved. Cell Counting Kit-8 is a colorimetric assay for determining viable cell numbers and can be used for cell proliferation assays and cytotoxicity assays. Cell Counting Kit-8 uses a tetrazolium salt, WST-8, which produces the water-soluble WST-8 formazan. Since this orange-colored formazan does not require dissolving, no solubilizing process is required. The results are obtained after 3 simple steps: (1) add, (2) incubate, and (3) read. This kit is applicable for 96-well microplate assays and can also be applied to high-throughput screening, such as for a 384-well microplate. WST-8 is not cell permeable, which results in low cytotoxicity. Therefore, it is possible to continue further experiments using the same cells after the assay [67].

### 4.8. Immunomodulatory Activity by Inhibition of IL-6 Gene Expression

IL-6 inhibition activity was assessed using a quantitative real-time PCR (qRT-PCR) method following RNA isolation. Cell lysis was performed by treating the harvested cells with Ribozol. The mixture was homogenized and incubated for 5 min. Lysate was transferred to microtubes and either stored at −80 °C or used immediately for extraction. For separation, the samples were thawed and briefly vortexed. Chloroform (200 μL per 1 mL Ribozol) was added, vortexed until homogenized, and then centrifuged at 12,000–16,000× *g* for 15 min. The aqueous phase (RNA) was collected carefully, avoiding the interphase and organic phase. For precipitation, isopropanol (equal to the volume of the aqueous phase) was added and vortexed. The mixture was incubated for 10 min, followed by centrifugation to form a white pellet. The supernatant was carefully removed. Washing was performed by washing the pellet with 70% ethanol (1 mL for 1 mL Ribozol), then vortexing and centrifuging. This wash step was repeated 2–3 times. For elution, the pellet was dried for 5–10 min and then dissolved in 20–50 μL NFW, followed by incubation at 55–60 °C for 10–15 min. For RNA concentration measurement, concentration was read using a microplate reader, ensuring a purity ratio of 1.7–2.0 and a suitable concentration for PCR (typically 100 ng/μL) [68].

## 5. Conclusions

The study highlights the successful extraction and isolation of kaempferol C_15_H_10_O_6_) from *Peronema canescens* leaves. Kaempferol has superior binding affinity and inhibition potential, supported by lower binding energy, inhibition constant (Ki), and stable interactions, including hydrogen bonding and π-π interactions. The molecular dynamics simulations further validated kaempferol’s stability and favorable interaction profile, as indicated by the low RMSD and RMSF values. Additionally, the MM-PBSA analysis showed kaempferol has strong Van der Waals and electrostatic interactions, reinforcing its binding affinity. The in vitro study shows that kaempferol exhibited significant inhibition of IL-6 gene expression in LPS-induced RAW 264.7 cells, reducing levels more effectively than the control. These results underscore kaempferol’s potential as a promising immunomodulatory agent targeting IL-6-related pathways.

## Figures and Tables

**Table 1:** Comparison of NMR data for kaempferol and the isolated compound.

No	Isolated Compound		Kaempferol [28]	
	δ_C_, Type	δ_H_, Mult. (*J* in Hz)	δ_C_, Type	δ_H_, Mult. (*J* in Hz)
2	146.7, C		146.9, C	
3	135.6, C		136.5, C	
4	175.8, C		176.5, C	
5	160.6, C		157.7, C	
6	98.1, CH	6.19, d (2.0)	98.9, CH	6.21, d (2.0)
7	163.8, C		165.0, C	
8	93.4, CH	6.44, d (2.0)	94.3, CH	6.43, d (2.0)
9	156.0, C		160.1, C	
10	102.9, C		103.9, C	
1′	121.6, C		123.1, C	
2′/6′	129.4, CH	8.05, d (9.0)	130.3, CH	8.11, d (8.5)
3′/5′	115.3, CH	6.93, d (9.0)	116.1, CH	6.93, d (8.5)
4′	159.1, C		161.9, C	
3-OH		9.43, s		9.30, s
5-OH		12.50, s		12.60, s
7-OH		10.80, s		10.90, s
4′-OH		10.13, s		10.20, s

**Table 2:** Result of molecular docking simulation with human Interleukin-6 (PDB ID: 1ALU) with TLA and kaempferol.

No	Compound	∆G kcal/mol	Ki (μM)	Types of Ligan Bonds and Interactions with Amino Acids			
				Hydrogen	Pi-Sigma	Pi-Alkyl	Pi-Amides
1	TLA	−5.90	47.35	Gln175, Arg182, Arg179	-	-	-
2	Kaempferol	−5.98	41.28	Gln175, Asp34	Leu33	Lys171	Ile36

**Table 3:** Pharmacokinetic properties and toxicity predictions of kaempferol.

Compound	Pharmacokinetic Prediction				Toxicity Prediction
	Absorption Caco-2 log Papp in 10^−6^ cm/s)	Distribution BBB (BB log)	Metabolism (CYP2D6) (Yes/No)	Excretion (Total Clearance) (Log mL/min/kg)	Ames (Yes/No)
Kaempferol	−5.26	−2.75	Not	5.81	Not

**Table 4:** Analysis of hydrogen bonds over 100 ns period using MD trajectories from all systems.

System	H-Bond Acceptors (res@atom)	H-Bond Donor (res@atom)	Fraction	Avg. Distance (Å)	Avg. Angle (◦)
TLA	TLA_158@O	Arg_155@H	0.10	28.07	1.586.07
	TLA_158@O	Arg_155@H	0.09	28.02	1.565.14
	TLA_158@O	Arg_12@H	0.09	28.03	1.606.04
	TLA_158@O	Arg_155@H	0.08	28.39	1.576.96
	TLA_158@O	Arg_152@H	0.07	28.48	1.574.45
	TLA_158@O	Arg_152@H	0.07	28.43	1.570.53
Kaempferol	Phe_98@O	Kaempferol@H	0.20	28.60	1.501.65
	Kaempferol@O	Arg_6@H	0.01	29.16	1.492.99
	Arg_6@O	Kaempferol@H	0.00	28.70	1.569.58
	Kaempferol@O	Ser_3@H	0.00	28.93	1.492.76
	Kaempferol@O	Leu_1@H	0.00	28.72	1.469.966
	Gln_97@O	Kaempferol@H	0.0023	28.66	1.544.905
	Kaempferol@O	Gln_10@H	0.0023	28.68	1.493.293
	Leu_1@O	Kaempferol@H	0.0022	28.62	1.434.219
	Kaempferol@O	Leu_1@H	0.0022	28.69	1.464.555
	Kaempferol@O	Leu_1@H	0.0020	28.68	1.468.561
	Kaempferol@O	Lys_101@H	0.0018	28.63	1.552.401

**Table 5:** Relative binding energy complex receptor–ligand.

Energy Component	Bond Energy (kcal/mol)						
	∆G_VDW_	∆G_EL_	∆E_GB_	∆E_SURF_	∆G_GAS_	∆G_SOLV_	∆G_TOTAL_
TLA	−0.44	−37.06	36.68	−0.19	−37.50	36.50	−1.00
Kaempferol	−17.02	−293.16	295.06	−2.73	−310.18	292.33	−17.85

## Data Availability

All data supporting the findings of this study are provided within the manuscript.
